# Synergistic anticancer effect of combined crocetin and cisplatin on KYSE-150 cells via p53/p21 pathway

**DOI:** 10.1186/s12935-017-0468-9

**Published:** 2017-10-30

**Authors:** Sheng Li, Xiu-Yin Shen, Ting Ouyang, Yuhua Qu, Tao Luo, Hua-Qiao Wang

**Affiliations:** 1 Department of Anatomy and Neurobiology, Zhongshan School of Medicine, Sun Yat-sen University, Guangzhou, 510080 Guangdong China; 2 Pediatric Hematology and oncology, Affiliated Guangzhou Women and Children’s Hospital, Zhongshan School of Medicine, Sun Yat-sen University, Guangzhou, 510623 Guangdong China

**Keywords:** Esophageal cancer, Crocetin, Cisplatin, KYSE-150 cell, Apoptosis

## Abstract

**Background:**

More than 400,000 patients die from esophageal cancer annually. Considerable efforts have been made to develop new and effective treatments, one of which is directed toward herbal medication. Crocetin is a natural carotenoid dicarboxylic acid isolated from the Chinese herb saffron. We recently reported on the anticancer effects of saffron. This study aimed to determine whether crocetin combined cisplatin has synergistic effect in KYSE-150 cells and explore the underlying mechanism.

**Methods:**

KYSE-150 cells were treated with crocetin and/or cisplatin. The effects on cell viability, cell apoptosis, mitochondrial membrane potential (MMP), as well as the expression levels of PI3K/AKT, MAPKs, p53/p21, and apoptosis-related protein were evaluated. MTT assay, Annexin V-FITC/PI staining, Rh123 staining, and Western blot analysis were used.

**Results:**

The cell proliferation significantly decreased and cell apoptosis was induced with combined crocetin and cisplatin, compared with either crocetin only or cisplatin only. The outcome suggested that crocetin combined cisplatin has synergistic effects on inhibition of cell proliferation and pro-apoptotic effect of cisplatin on KYSE-150 cells. Disruption of MMP, upregulation of cleaved caspase-3 expression, and downregulation of Bcl-2 occurred in the group treated with combined treatment. No significant differences in p-PI3K, p-AKT, and MAPKs activity were indicated between combined treatment group and the individual treatment group. However, the expression levels of p53 and p21 were markedly higher in the combined treatment group than in the individual treatment group. The wild-type p53 inhibitor, PFT-α suppressed the overexpression of p53/p21 and the synergistic effect induced by the combination of crocetin and cisplatin.

**Conclusions:**

We concluded that crocetin combined with cisplatin exerts a synergistic anticancer effect by up-regulating the p53/p21 pathway.

## Introduction

Esophageal cancer (EC) ranks eighth among the cancers with the highest incidence rate worldwide. The reason is that prominent symptoms rarely appear until the advanced stages of the disease. About 20–30% of patients with EC have distant metastasis at the time of the initial diagnosis [1], which impedes the treatment of EC. The disease has a poor prognosis, with its 5-year survival rate of only 13% [2]. To lengthen survival in patients with EC, chemotherapy is considered the most important treatment. However, the toxicity of most chemotherapeutic agents to normal tissues presents a major obstacle to successful treatment. Therefore, a highly effective therapy with reduced side effects urgently needed.

Many previous studies proved that combining prescription medications and herbal medicine can improve anticancer properties and reduce side effects [3–5]. Saffron is a Chinese traditional herb. Crocetin, the major constituent of saffron, has drawn research interest for its multiple pharmacological effects, including anticancer properties [6, 7]. Cytotoxicity in various cancer cell lines, including human gastric cells [8], colon cancer cells [9], and cervical cancer cells [10] has been confirmed. Our previous studies [11] exerted a marked inhibitory effect on the growth of KYSE-150 cells. Cisplatin (DDP) combination therapy is considered the cornerstone of treatment of many cancers. However, after the initial use of this combined treatment, patients gradually became less sensitive to cisplatin, particularly after a long-term treatment or recurrence. Considerable efforts have been directed toward improving the therapeutic efficacy of cisplatin. Numerous studies combined an anticancer herb with cisplatin to enhance the therapeutic effect of cisplatin [12, 13], given that many herbs exhibit either no toxicity or reduced toxicity to humans. The present study aimed to investigate whether combined crocetin and cisplatin exerts a synergistic effect on KYSE-150 cells and explore its underlying mechanism by examining phosphatidylinositol 3-kinase (PI3K)-protein kinase B (AKT), mitogen-activated protein kinases (MAPKs), and p53/p21 in KYSE-150 cells, which significantly influence the occurrence, progression, and prognosis of tumors.

## Materials and methods

### Chemicals and reagents

Crocetin (C_20_H_24_O_4_, molecular weight, 328.4) and dimethyl sulfoxide (DMSO) were supplied by MP Biomedicals (Santa Ana, CA, USA). Crocetin was dissolved in DMSO, stored at − 20 °C, and diluted in a medium prior to each experiment. The final DMSO concentration did not exceed 0.1% throughout the study. Dulbecco’s Modified Eagle’s Medium (DMEM), penicillin, and streptomycin were purchased from Gibco (Gibco Life Technologies, Carlsbad, CA, USA). Pifithrin-α-HBr (ab120478) (PFT-α) was supplied by Abcam (Abcam, Cambridge, UK). 3-(4,5-dimethyl-2-thiazolyl)-2,5-diphenyl-2-*H*-tetrazolium bromide (MTT) was purchased from Sigma-Aldrich (St. Louis, MO, USA). Annexin V-FITC/PI Apoptosis Assay Kit was obtained from BestBio (BestBio Co., Ltd, Shanghai, China). Bicinchoninic Acid Protein Assay Kit was purchased from Beyotime Institute of Bioengineering (Jiangsu, China). Horseradish peroxidase-conjugated goat anti-rabbit antibodies were obtained from Wuhan Boster Biological Technology, Ltd. (Wuhan, Hubei, China).

### Cell culture

The esophageal squamous carcinoma KYSE-150 cell line was purchased from Japanese Collection of Research Bioresources Cell Bank (JCRB, Osaka, Japan). The KYSE-150 cells were cultured in DMEM supplemented with 10% fetal bovine serum (Zhejiang Tianhang Biological Technology Stock Co., Ltd., Zhejiang, China), 100 units/mL of penicillin and 100 μg/mL of streptomycin cells were maintained at an atmosphere of 5% CO_2_ and 95% air at 37 °C. When cells in the logarithmic growth phase were used for experiments, each test was performed at least in triplicate.

### MTT assay to assess cell proliferation

The effect of crocetin and cisplatin on cell viability was measured by MTT assay as described in a previous study [14]. KYSE-150 cells were seeded in 96-well plates at a density of 6000 cells/well in complete DMEM and then incubated at 37 °C. After 24 h, the cells were incubated with varying concentrations of cisplatin (0.25, 0.5, 1, 2, 4, and 8 μg/mL) or combined 200 μmol/L of crocetin and 2 μg/mL of cisplatin for 24, 48, and 72 h. After incubation, the medium was aspirated carefully, and the 96-well plate was gently washed with phosphate buffered saline (PBS). Cells were then incubated with MTT. After 4 h, the supernate was discarded, and formazan was dissolved in DMSO. The absorbance of each well was measured at 570 nm in a microplate reader (Tecan Austria GmbH, Grödig, Austria).

### Morphologic detection of apoptosis

Cell apoptosis induced by crocetin and cisplatin was determined using the Annexin V-FITC/PI apoptosis kit in accordance with the manufacturer’s instructions. Cells were plated in a 12-well plate at a density of 1.2 × 10^5^ cells/well and then treated with different agents (control, 200 μmol/L of crocetin, 0.2 μg/mL of cisplatin, 200 μmol/L of crocetin + 0.2 μg/mL of cisplatin) for 48 h. The cells were subsequently washed thrice with ice-cold PBS and incubated with 5 μL Annexin V-FITC, 10 μL of PI mixed in a 400 μL of buffer solution for 15 min at 37 °C in a humidified atmosphere in the dark. The plate was gently washed three times with PBS. A fluorescent apoptotic cell morphology was observed under an inverted phase contrast microscope and then photographed. Annexin V- or PI-positive cells were identified as apoptotic cells. The numbers of apoptotic cells were expressed as percentages of total cells of each field, and at least three fields were calculated [15, 16].

### Measurement of mitochondrial membrane potential (MMP)

The MMP was measured by inverted-phase contrast microscopy by using the fluorescent dye Rhodamine 123 (Rh123). KYSE-150 cells were plated into 12-well plates and then exposed to crocetin or/and cisplatin for 48 h. After incubation, cells were washed with PBS three times and then incubated with 2 μmol/L Rh123 for 30 min at 37 °C in a humidified atmosphere in the dark. The plate was gently washed three times with PBS. Images of the cells were finally obtained under an inverted phase contrast microscope and analyzed using Image-Pro Plus 6.0.0.260 (Media Cybernetics, USA).

### Western blot analysis

After treatment with different drugs for 48 h, the KYSE-150 cells were harvested and lyzed in an ice-cold lysis buffer (1× PBS; 1% NP40; 0.1% SDS; 5 mm EDTA; 0.5% sodium deoxycholate; 1% PMSF) for 30 min. The lysate was cleared by centrifugation at 14,000×*g* for 15 min at 4 °C. The supernatant extract was collected and quantified for protein by using the BCA protein assay kit. Equal quantities of cell protein were subjected to electrophoresis in 10 or 12% sodium dodecyl sulfate–polyacrylamide gel electrophoresis (SDS-PAGE). The protein was then transferred to polyvinylidene difluoride (PVDF) membranes and subsequently blocked using 5% bovine serum albumin for 1 h at room temperature and was then incubated overnight with primary antibodies at 4 °C. The blots were washed three times with Tris-buffered saline (Guangzhou Whiga Biotechnology Co., Ltd., Guangzhou, China) containing 0.05% Tween-20 (Wuhan Boster Biological Technology, Ltd.) and then incubated with horseradish peroxidase-conjugated anti-rabbit antibodies for 1 h at room temperature. The protein bands were detected by electrochemiluminescence, and the band intensity was measured using ImageJ 1.46r (National Institutes of Health, Bethesda, MA, USA). Each experiment was repeated at least three times. The Western blot antibodies used are listed in Table 1.

**Table 1:** Antibodies used in Western blot analysis

Antibody	Type	Dilution	Source
PI3K	mAb	1:1000	Cell signaling
p-PI3K	mAb	1:2000	Cell signaling
AKT	mAb	1:1000	Cell signaling
p-AKT	mAb	1:1000	Cell signaling
ERK1/2	mAb	1:1000	Cell signaling
p-ERK1/2	mAb	1:1000	Cell signaling
JNK	mAb	1:1000	Cell signaling
p-JNK	mAb	1:1000	Cell signaling
p38	mAb	1:1000	Cell signaling
p-p38	mAb	1:1000	Cell signaling
p53	pAb	1:1000	Cell signaling
p21	mAb	1:1000	Cell signaling
Bax	mAb	1:1000	Abcam
Bcl-2	mAb	1:1000	Abcam
Cleaved caspase-3	mAb	1:2000	Cell signaling
GAPDH	mAb	1:1000	Millipore

### Statistical analysis

All data were collected from at least three experiments and expressed as mean ± SEM. The differences among groups—control, crocetin, cisplatin, and combination of agents—were analyzed by one-way ANOVA, followed by the least significant difference post hoc test in SPSS 16. (SPSS, Inc., Chicago, IL, USA). p < 0.05 was considered statistically significant.

## Results

### Effect of combined crocetin and cisplatin on proliferation of KYSE-150 cells

**Fig. 1:**
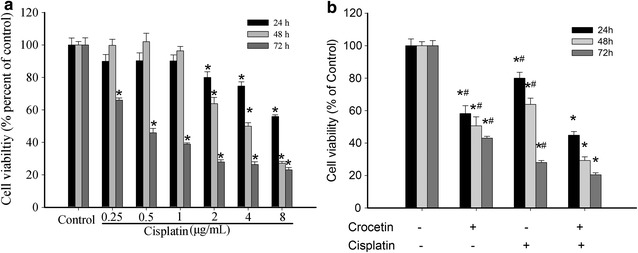
Effect of combined crocetin and cisplatin on cell proliferation of KYSE-150 cells. Cell viability was measured by MTT after incubation with different agents for 24, 48, and 72 h. **a** Cell viability of KYSE-150 cells treated with varying concentrations of cisplatin. **b** Cell viability of KYSE-150 cells treated with combined 200 μmol/L crocetin and 2 μg/L cisplatin. *p < 0.05 compared with the control group; ^#^p < 0.05 compared with the combined crocetin and cisplatin group

As shown in Fig. 1, in 24 or 48 h, proliferation of KYSE-150 cells could not be inhibited until 2 μg/mL of cisplatin was applied. Thus, we used the minimal effective concentration of 2 μg/mL in the study. To evaluate whether the combined treatment of crocetin and cisplatin exerted a synergistic effect on KYSE-150 cells, we determined the cell viability by using 200 μmol/L of crocetin [11] and 2 μg/mL of cisplatin, individually or combined, to treat the KYSE-150 cells. Compared with the individual treatments (Table 2), the combination of 200 μmol/L of crocetin and 2 μg/mL of cisplatin significantly inhibited the cell viability in a time-dependent manner (p < 0.05). This difference suggested that crocetin combined with cisplatin exerts a synergistic effect on the viability of the KYSE-150 cells.

**Table 2:** The synergistic effect of crocetin and cisplatin on proliferation of KYSE-150 cells at different times

Time (h)	Group	Group	Mean difference (%)	*p*	95% confidence interval (%)	
24	C + D	Crocetin	− 13.31^a^	0.042	− 26.05	− 0.56
		DDP	− 36.17^a^	0.000	− 48.91	− 23.43
48	C + D	Crocetin	− 21.35^a^	0.004	− 33.97	− 8.73
		DDP	− 37.38^a^	0.000	− 50.00	− 24.76
72	C + D	Crocetin	− 19.80^a^	0.000	− 24.03	− 15.57
		DDP	− 4.73^a^	0.032	− 8.97	− 0.50

^a^The mean difference is significant at the 0.05 level

### The synergistic effect of combined crocetin and cisplatin induced cytotoxicity in KYSE-150 cells

**Fig. 2:**
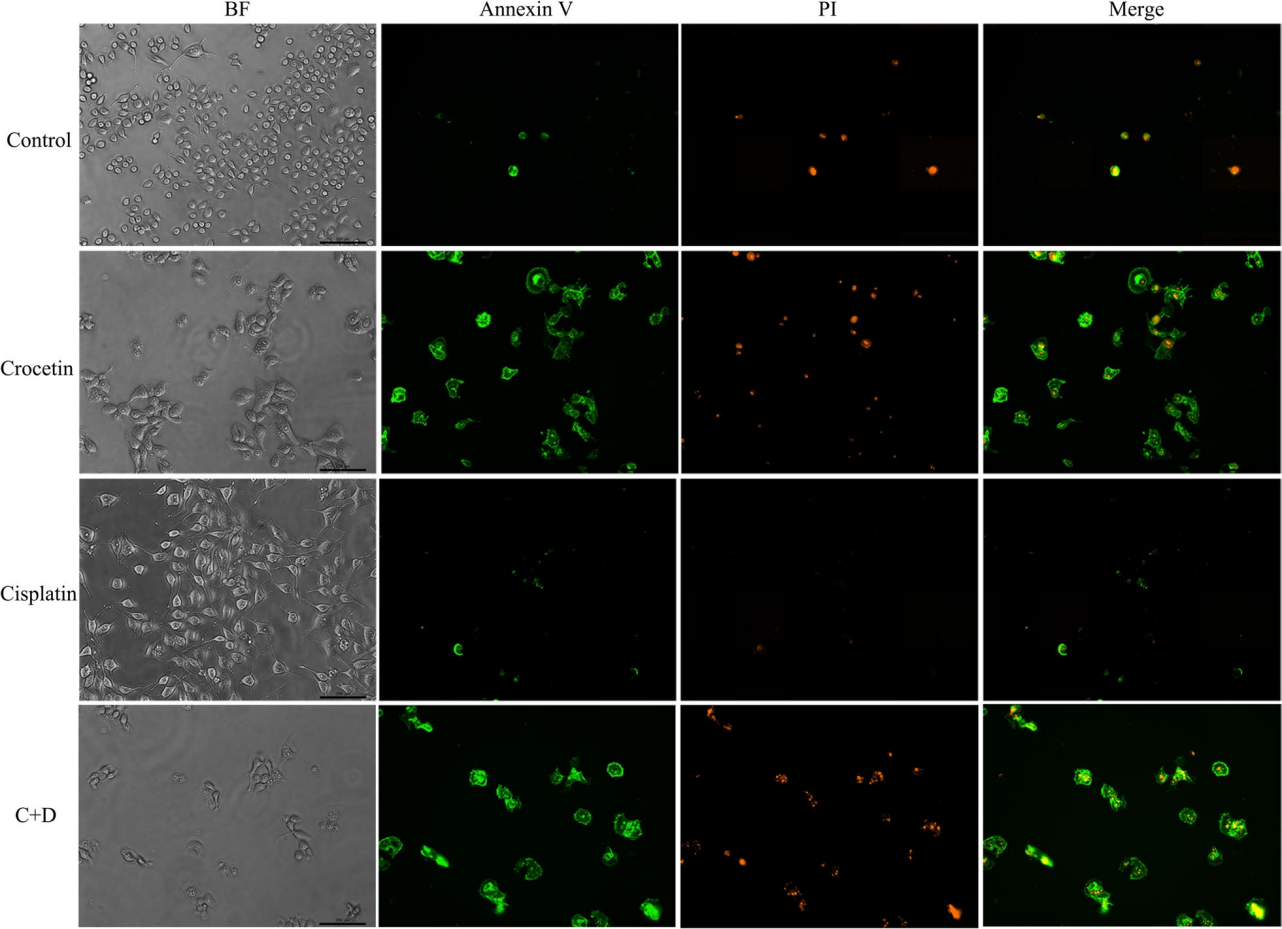
Morphological changes of KYSE-150 cells after incubation with crocetin and/or cisplatin. KYSE-150 cells were untreated (control) or treated with 200 μmol/L of crocetin, 2 μg/L of cisplatin, and 200 μmol/L of crocetin + 2 μg/L cisplatin (C + D) for 48 h. Morphological changes were observed under an inverted phase-contrast microscope (BF) or stained with an Annexin V/PI solution under a fluorescence microscope. Scale bar = 100 μm

Figure 2 shows that the normal KYSE-150 cells are attached to the dish, polygon-like with distinct cell borders under the inverted phase-contrast microscope. However, after the cells were treated with crocetin or cisplatin for 48 h, the cell morphology markedly changed: the cells were significantly reduced in number, appeared shrunk and round, and became loosely arranged. The cells in the combined treatment group were also more seriously damaged than the cells in the single treatment group. Annexin V-FITC/PI staining also indicated that cell apoptosis was more serious after the addition of crocetin and/or cisplatin. The percentages of apoptotic cells were (19.87 ± 3.48), (9.87 ± 0.20), and (4.99 ± 0.38) % in the combined treatment group, crocetin-only group, and cisplatin-only group, respectively. Cell apoptosis was markedly higher in the combined treatment group than in the single treatment group (p < 0.05). This difference suggested that combined crocetin and cisplatin could induce more apoptosis in KYSE-150 cells.

### The synergistic effect of crocetin on cisplatin decreased MMP in KYSE-150 cells

**Fig. 3:**
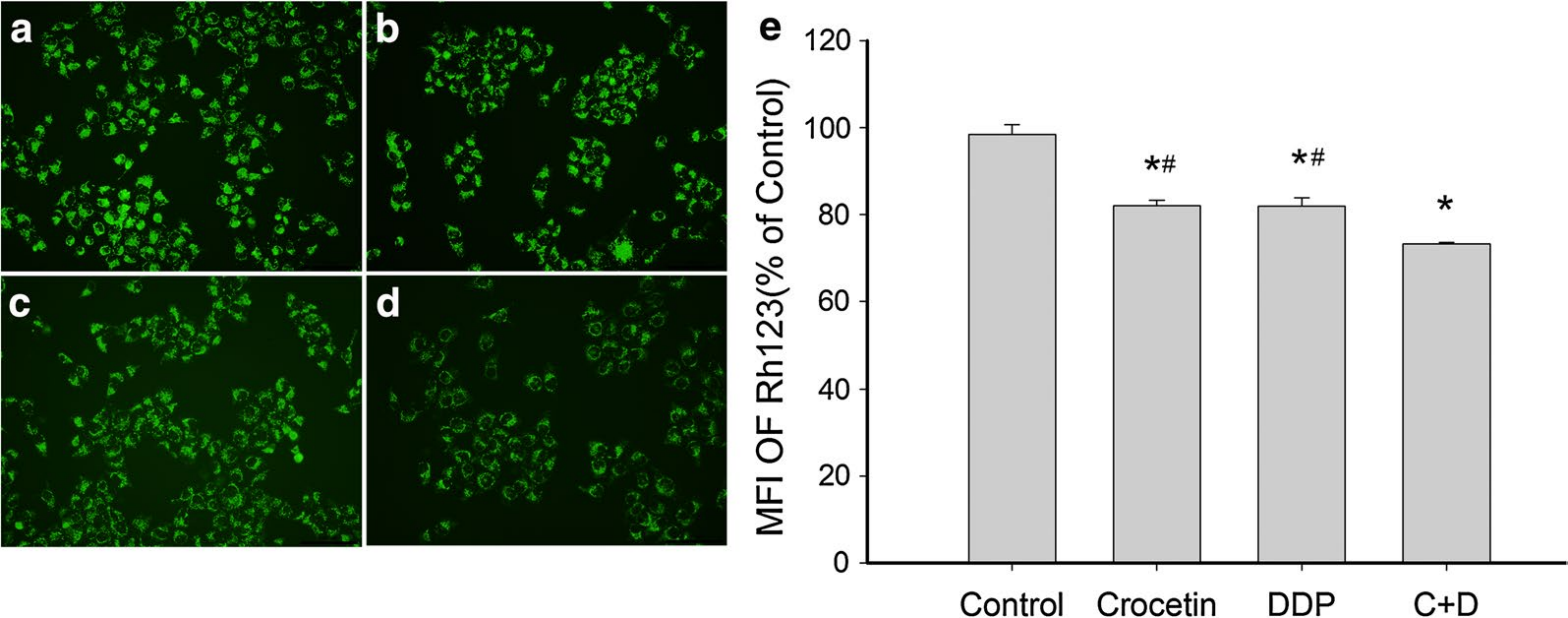
Mitochondrial depolarization was involved in apoptosis induced by combined crocetin and cisplatin treatment. KYSE-150 cells were treated with crocetin or/and cisplatin for 48 h, stained with Rh123 solution, and observed under a fluorescence microscope. **a** Control; **b** 200 μmol/L of crocetin; **c** 2 μg/L of cisplatin; **d** 200 μmol/L of crocetin + 2 μg/L of cisplatin. **e** Mean fluorescence intensity analysis. Each bar represents the mean ± SEM, *p < 0.05 compared with the control group; ^#^ *p* < 0.05 compared with the combined crocetin and cisplatin group. Scale bar = 100 μm

Disruption of MMP occurred in the early phase of mitochondria-mediated apoptosis. The effect of crocetin or/and cisplatin on the change in MMP in the KYSE-150 cells was detected by the alterations in fluorescence intensity with Rh 123 treatment. The green fluorescence intensity weakened after treatments were applied, as shown in Fig. 3. The mean fluorescence intensity of Rh123 was (73.27 ± 0.35) % in the combined treatment treated group, (81.98 ± 1.94) % in the cisplatin-only group, and (82.12 ± 1.14) % in the crocetin-only group. The fluorescence intensity was markedly lower in the combined treatment group than in the single treatment group (p < 0.05). These results indicated that the combination of crocetin and cisplatin could synergistically enhance the disruption of MMP in KYSE-150 cells.

### Combination of crocetin and cisplatin regulated the expression of apoptosis-related proteins in KYSE-150 cells

**Fig. 4:**
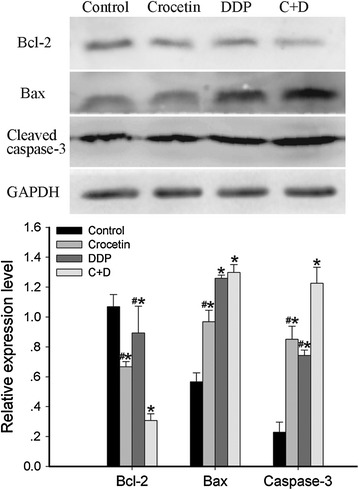
Effect of crocetin and cisplatin on the expression of apoptosis-related proteins. KYSE-150 cells treated with different agents for 48 h. The expression levels of Bcl-2, Bax, and cleaved caspase-3 were detected by Western blot analysis. DDP, 2 μg/L of cisplatin; C + D, 200 μmol/L of crocetin + 2 μg/L of cisplatin. Each bar represents mean ± SEM, *p < 0.05 compared with the control group; ^#^ *p* < 0.05 compared with the combined crocetin and cisplatin group

To explore the mechanisms underlying the apoptosis induced by the combined treatment of crocetin and cisplatin, the expression levels of pro-apoptotic and anti-apoptotic protein were evaluated by Western blot analysis. As shown in Fig. 4, the expression levels of Bax and cleaved caspase-3 in both crocetin and cisplatin groups were higher and the Bcl-2 levels were lower than those in the control group (p < 0.05). More changes were found in the combined treatment group. The expression of cleaved caspase-3 was markedly higher (p < 0.05), and the Bcl-2 level was markedly lower (p < 0.05) in the combined treatment group than in the single treatment group. However, the Bax level in the combined treatment group was significantly (p < 0.05) increased with control and crocetin but not significantly with cisplatin. These results indicated that the combination of crocetin and cisplatin could increase the expression of apoptosis-related proteins in KYSE-150 cells.

### Effect of crocetin and cisplatin on PI3K/AKT pathway in KYSE-150 cells

**Fig. 5:**
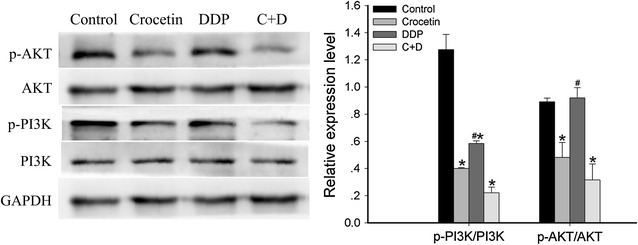
Effects of crocetin and cisplatin on PI3K/AKT pathways in KYSE-150 cells. The expression levels of p-PI3K, PI3K, p-AKT, and AKT in KYSE-150 cells were untreated (control) or treated with 200 μmol/L of crocetin, 2 μg/L of cisplatin (DDP), and 200 μmol/L of crocetin + 2 μg/L of cisplatin (C + D) for 48 h, as determined by Western blot analysis. GAPDH was used as loading control. *p < 0.05 compared with the control group; ^#^p < 0.05 compared with the combined crocetin and cisplatin group

Numerous studies have reported that the overactivated PI3K/AKT pathway influences carcinogenesis and chemoradiotherapy resistance. Thus, we detected the activation of the PI3K/AKT pathway in KYSE-150 cells. As shown in Fig. 5, the combined treatment group significantly reduced the expression levels of p-PI3K (p < 0.001) and p-AKT (p < 0.01) relative to the control group; however, the combined treatment group failed to show a significant reduction relative to the crocetin-only group. These results suggested that the activation of the PI3K/AKT signaling pathway was not required for the effects of combined crocetin and cisplatin.

### Effect of crocetin and cisplatin on MAPKs expression in KYSE-150 cells

**Fig. 6:**
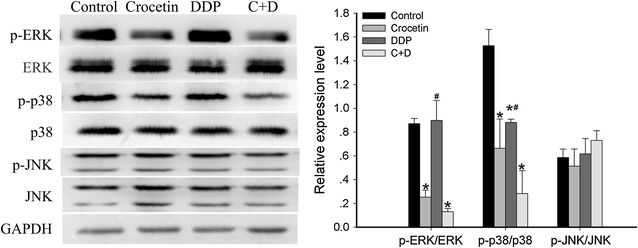
Effects of crocetin and cisplatin on MAPK pathways in KYSE-150 cells. The expression levels of p-ERK1/2, ERK1/2, p-p38, p38, p-JNK, and JNK in KYSE-150 cells were untreated (control), or treated with 200 μmol/L of crocetin, 2 μg/L of cisplatin (DDP), and 200 μmol/L of crocetin + 2 μg/L of cisplatin (C + D) for 48 h, as determined by Western blot analysis. GAPDH was used as a loading control. *p < 0.05 compared with the control group; ^#^p < 0.05 compared with the combined crocetin and cisplatin group

To determine the relevance of MAPKs activation in apoptosis induced by combined crocetin and cisplatin, we analyzed the expression of MAPKs in KYSE-150 cells. As shown in Fig. 6, the activation of ERK1/2 in the combined treatment group was significantly inhibited relative to that in the control group (p < 0.001) and the cisplatin-only group (p < 0.001); however, the combined treatment group showed no significant reduction compared with the crocetin group. The activation of P38 in the combined treatment group was markedly inhibited relative to that in the cisplatin group (p < 0.05) and the control group (p < 0.01); however, the combined treatment group showed no difference from the crocetin group. In addition, both combined and individual treatments showed no significant effect on the activation of c-jun N-terminal kinase (JNK). These findings indicated that the synergitsic effect of crocetin on cisplatin was not attributable to the MAPKs pathway.

### Crocetin combined with cisplatin upregulated p53/p21 expression in KYSE-150 cells

**Fig. 7:**
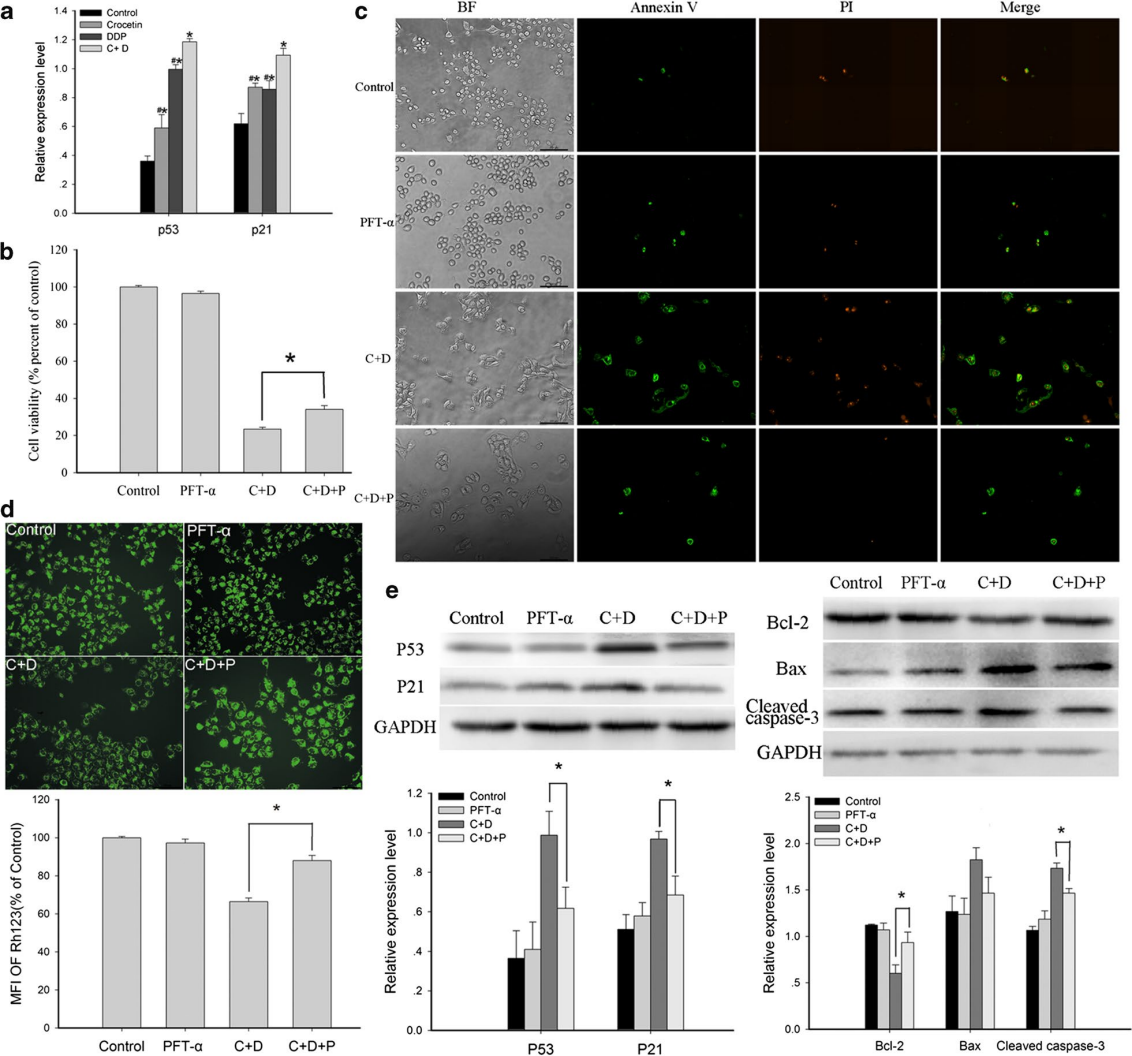
P53/p21 pathway was involved in the synergistic anticancer effect of crocetin and cisplatin. **a** Expression levels p53, p21 in KYSE-150 cells, as determined by Western blot analysis. **b** Cell viability in the presence of 2 μM PFT-α (p53 inhibitor) for 48 h. **c** Morphological changes after adding PFT-α. **d** MMP changes after the application of PFT-α. **e** Western blot analysis of p53, p21, and apoptosis-related protein expression after adding PFT-α. C + D, 200 μmol/L of crocetin + 2 μg/L of cisplatin, C + D + P, 200 μmol/L of crocetin + 2 μg/L of cisplatin + 2 μmol/L PFT-α. *p < 0.05 compared with the control group; ^#^p < 0.05 compared with the combined crocetin and cisplatin treatment group

To explore the underlying mechanism of the anticancer effect of crocetin and cisplatin, we measured the expression levels of p53 and p21 in KYSE-150 cells. As shown in Fig. 7a, p53 expression was markedly higher in both the crocetin-only group (p < 0.01) and the cisplatin-only group (p < 0.001) than in the control group. In addition, p53 expression was higher in the combined treatment group than in the single treatment group (compared with crocetin, p < 0.001; compared with cisplatin, p < 0.05); p21 expression was also higher in both the crocetin-only group (p < 0.01) and the cisplatin-only group (p < 0.05) than in the control group. In addition, p21 expression was higher in the combined treatment group than in the single treatment group (compared with crocetin, p < 0.05; compared with cisplatin, p < 0.05). These suggested that the synergistic anticancer effects of crocetin and cisplatin has a close link with p53/p21 pathway.

### Pifithrin-α partially reverted the synergistic effect of combined crocetin and cisplatin

To verify the function of the p53/p21 pathway in the synergistic effect of combined crocetin and cisplatin, we treated cells with or without pifithrin-α, a wild- type p53 inhibitor. As shown in Fig. 7b, after pifithrin-α was added, cell viability was significantly increased (p < 0.001) from (23.42 ± 1.01) to (34.03 ± 2.10) %. Cell apoptosis was also significantly reduced (p < 0.001) from (14.07 ± 0.60) to (8.57 ± 0.44) % after pifithrin-α was added (Fig. 7c). As shown in Fig. 7d, MMP was also higher (87.94 ± 2.79) % in the combined pifithrin-α, crocetin, and cisplatin treatment group than in the combined crocetin and cisplatin group (66.44 ± 1.84) % (p < 0.001). Meanwhile, the mitochondrial apoptosis-related protein expression was also altered after pifithrin-α was added (Fig. 7e). Compared with that of the combined treatment crocetin and cisplatin group, Bcl-2 expression was higher (p < 0.05) and cleaved caspase-3 expression was lower (p < 0.05) in the combined crocetin, cisplatin, and pifithrin-α group. In addition, the expression levels of p53 and p21 in the combined cisplatin and crocetin group were suppressed after pifithrin-α was added; this result suggested that combined crocetin and cisplatin exerted a synergistic anticancer effect via the activation of the p53-dependent pathway.

## Discussion

Cisplatin is a basement chemotherapy for EC, and initial cisplatin responsiveness is highly effective. However, some patients can eventually fall into relapse with cisplatin resistance over time. In addition, the side effect of cisplatin is another issue in cisplatin treatment in the clinical setting. Thus, effective chemotherapy with reduced side effects is urgently needed for EC patients. A growing number of studies have focused on whether the combination of prescription medications and anticancer herbs could achieve a synergistic effect and reduce side effects [12, 13]. Crocetin, a sort of carotenoid, has reportedly exhibited biomedical properties, including anticancer properties as well as less or non-toxicity to normal cells [8, 17, 18]. In the present study, we investigated whether crocetin combined cisplatin has synergistic effect in KYSE-150 cells and explored the underlying mechanism. The results we obtained indicated that the combination of crocetin and cisplatin significantly inhibited cell proliferation and induced cell apoptosis in KYSE-150 cells. Mechanistically, this synergistic effect exhibits a close association with the up regulation of p53/p21 expression as well as the activation of mitochondria- mediated apoptosis pathway, as manifested by the overexpression of p53 and p21, loss of MMP, upregulation of Bcl-2, and downregulation of cleaved caspase-3. The synergistic effect was also partially blocked after pifithrin-α was added.

A previous study has shown that tumorigenesis has a close relationship with the cell signaling pathway. In this study, we analyzed the expression of PI3K/AKT, MAPKs, and p53/p21 pathways in KYSE-150 cells. The PI3K/AKT pathway is an intracellular signaling pathway that significantly affects cell proliferation. The phosphorylated PI3K can activate AKT. The activated AKT can further phosphorylate BAD on Ser-136 and prompt it to dissociate from the Bcl-2/Bcl-xl complex, ultimately resisting cell apoptosis. The PI3K/AKT pathway has been proven to be related to the occurrence, progression, and prognosis of EC, as well as resistance to chemotherapy and radiotherapy [19–22]. Moreover, the inhibitor of the PI3K/Akt pathway significantly suppressed cell proliferation and induced cell apoptosis via suppressed Bcl-xl expression. Thus, the PI3K/AKT pathway could be potentially used for cancer treatment. Our results indicated that cisplatin decreased p-PI3K but not p-AKT expression (Fig. 5). These results may be associated with the resistance of KYSE-150 cells to cisplatin [23]. PI3K/AKT expression was significantly lower in the combined crocetin and cisplatin group than in the cisplatin-only group; however, the reduction was not as much in the crocetin-only group. Thus, we concluded that the PI3K/AKT signaling pathway does not induce the response for combined crocetin and cisplatin to exert a synergistic effect on the KYSE-150 cells.

In addition, we determined the expression of mitogen-activated protein kinases in KYSE-150 cells. MAPKs include the extracellular signal-regulated kinase 1 and 2 (ERK1/2), JNK, and p38, which are involved in cell proliferation, differentiation, mitosis, and apoptosis. The activation of ERK1/2 phosphorylated Bim and/or Bad reduced the sensitivity of cells to apoptosis and promoted cell proliferation [24]. The phosphorylated p38 and JNK could induce cell apoptosis; however, several studies found that p38 could also induce cell proliferation and anti-apoptosis, particularly in an inflammatory environment [25, 26]. Overexpression of ERK1/2, p38, and JNK occurred in esophageal cancer patients [27–29]. However, suppression of the expression of ERK1/2 and p38 induced prominent cell apoptosis in carcinoma cells [30, 31]. The expression of Aquaporin 5 was positively correlated with drug resistance in colon cancer, and silencing of Aquaporin 5 suppressed p38 MAPK signaling and improved drug resistance in colon cancer cells. In addition, using a p38 inhibitor could also exert a similar effect [32]. Therefore, the recovery of the normal expression of MAPKs is important for EC treatment. In the current study, the combined crocetin and cisplatin treatment markedly down-regulated the levels of p-ERK1/2 and p-p38 but showed no significant decrease compared with the crocetin-only treatment (Fig. 6). These results suggested that the synergistic effect of crocetin and cisplatin was not attributable to the MAPK pathway either.

Finally, we explored the p53/p21 pathway in the KYSE-150 cells. P53 is encoded by the tumor suppressor gene p53, which plays an important role in the regulation of cell cycle, cell apoptosis, and inhibition of angiogenesis. P21, widely known as cyclin-dependent kinase inhibitor 1, regulates cell cycle progression. Activated p21 could induce the arrest of cell growth. P21 is highly controlled by p53. An increasing number of evidence confirmed that the occurrence of EC exhibits a close correlation with high mutant p53 expression and low p21 expression [33]. However, mutant p53 lost it normal function. Thus, restoring the normal function of p53 is key to cancer treatment. The results of our study indicated that the expression levels of p53 and p21 were higher with the combined crocetin and cisplatin treatment than with the single treatments. Adding pifithrin-α, a wild-type p53 inhibitor, partially restored cell proliferation and rescued the cells from death. Moreover, p53 induced cell apoptosis via up-regulated expression of Bid, another pro-apoptotic protein in the Bcl-2 family. Overexpression of p53 could trigger mitochondria-mediated apoptosis pathway and induce the disruption of MMP, downregulation of Bcl-2, cyto-C release, and activation of caspase-3. Our results indicated that compared with the single treatments, combined crocetin and cisplatin markedly increased cleaved caspase-3 expression and decreased Bcl-2 expression. However, adding pifithrin-α partially blocked this mitochondria-mediated apoptosis pathway. Therefore, we concluded that the synergistic effect of combined crocetin and cisplatin was closely associated with the p53/p21 pathway.

## Conclusion

To date, our study is the first to demonstrate that the synergistic effects of crocetin and cisplatin on inhibition of cell proliferation and pro-apoptotic effect in KYSE-150 cells. The underlying mechanism was associated with the up regulation of the p53/p21 pathway and induction of the mitochondria-mediated apoptosis pathway, as shown by the disruption of MMP. Crocetin combined cisplatin also decreased Bcl-2 expression and increased cleaved caspase-3 expression. However, as our study examined only one cell line, more cell lines and in vitro experiments should be tested. In addition, the mechanism under the synergistic effect of crocetin and cisplatin still need further investigate. How to modulate p53/p21 pathway to obtain the synergistic effect? Such as MDM2, which is an important regulator of the p53 and function as ubiquitin ligase that recognizes the N-terminal trans-activation domain and as an inhibitor of p53 transcriptional activation. Nevertheless, our study clearly indicated that combined crocetin and cisplatin exhibits a synergistic effect and can provide a potential treatment for EC patients.

## Abbreviations

DDP: cisplatin
DMEM: Dulbecco’s Modified Eagle’s Medium
DMSO: dimethyl sulfoxide
EC: esophageal cancer
ERK1/2: extracellular signal-regulated kinase 1 and 2
JNK: c-jun N-terminal kinase
MAPKs: mitogen-activated protein kinases
MMP: mitochondrial membrane potential
MTT: 3-(4,5-dimethyl-2-thiazolyl)-2,5-diphenyl-2-*H*-tetrazolium bromide
PBS: phosphate buffered saline
PFT-α: pifithrin-α-HBr
PI3 K: phosphatidylinositol 3-kinase
PVDF: polyvinylidene difluoride
Rh123: rhodamine 123
SDS-PAGE: sodium dodecyl sulfate–polyacrylamide gel electrophoresis
